# The overall health and risk factor profile of Australian Aboriginal and Torres Strait Islander participants from the 45 and up study

**DOI:** 10.1186/1471-2458-13-661

**Published:** 2013-07-17

**Authors:** Lina Gubhaju, Bridgette J McNamara, Emily Banks, Grace Joshy, Beverley Raphael, Anna Williamson, Sandra J Eades

**Affiliations:** 1 Baker IDI Heart and Diabetes Institute, Preventative Health, 75 Commercial Road, 3004, Melbourne, VIC, Australia; 2 National Centre for Epidemiology and Population Health, The Australian National University, Building 62, 0200, Canberra, ACT, Australia; 3 Psychological and Addiction Medicine, The Australian National University, Building 42, 0200, Canberra, ACT, Australia; 4 The Sax Institute, Haymarket, K617, NSW 1240, Sydney, Australia; 5 School of Public Health, The University of Sydney, A27 Edward Ford Building, 2006, Sydney, NSW, Australia

**Keywords:** Aboriginal Australians, Torres Strait Islanders, 45 and Up study

## Abstract

**Background:**

Despite large disparities in health outcomes between Aboriginal and non-Aboriginal Australians, detailed evidence on the health and lifestyle characteristics of older Aboriginal Australians is lacking. The aim of this study is to quantify socio-demographic and health risk factors and mental and physical health status among Aboriginal participants from the 45 and Up Study and to compare these with non-Aboriginal participants from the study.

**Methods:**

The 45 and Up Study is a large-scale study of individuals aged 45 years and older from the general population of New South Wales, Australia responding to a baseline questionnaire distributed from 2006–2008. Odds ratios (OR) and 95% confidence intervals (CI) of self-reported responses from the baseline questionnaire for Aboriginal versus non-Aboriginal participants relating to socio-demographic factors, health risk factors, current and past medical and surgical history, physical disability, functional health limitations and levels of current psychological distress were calculated using unconditional logistic regression, with adjustments for age and sex.

**Results:**

Overall, 1939 of 266,661 45 and Up Study participants examined in this study identified as Aboriginal and/or Torres Strait Islander (0.7%). Compared to non-Aboriginal participants, Aboriginal participants were significantly more likely to be: younger (mean age 58 versus 63 years); without formal educational qualifications (age- and sex- adjusted OR = 6.2, 95% CI 5.3-7.3); of unemployed (3.7, 2.9-4.6) or disabled (4.6, 3.9-5.3) work status; and with a household income < $20,000/year versus ≥ $70,000/year (5.8, 5.0-6.9). Following additional adjustment for income and education, Aboriginal participants were significantly more likely than non-Aboriginal participants to: be current smokers (2.4, 2.0-2.8), be obese (2.1, 1.8-2.5), have ever been diagnosed with certain medical conditions (especially: diabetes [2.1, 1.8-2.4]; depression [1.6, 1.4-1.8] and stroke [1.8, 1.4-2.3]), have care-giving responsibilities (1.8, 1.5-2.2); have a major physical disability (2.6, 2.2-3.1); have severe physical functional limitation (2.9, 2.4-3.4) and have very high levels of psychological distress (2.4, 2.0-3.0).

**Conclusions:**

Aboriginal participants from the 45 and Up Study experience greater levels of disadvantage and have greater health needs (including physical disability and psychological distress) compared to non-Aboriginal participants. The study highlights the need to address the social determinants of health in Australia and to provide appropriate mental health services and disability support for older Aboriginal people.

## Background

In an era where people in developed nations such as Australia are, on average, living longer and healthier lives, Australian Aboriginal people still have a considerably shorter life expectancy and experience a significantly higher burden of disease and disability throughout the life course, compared to non-Aboriginal Australians. This has partly been attributed to the historical consequences of colonisation of Australia that resulted in loss of traditional lifestyles, culture, and self determination for Aboriginal people [1]. Racism, discrimination, marginalisation from society and on-going consequences of forced removal of children from Aboriginal families [2] have no doubt influenced the current social, emotional and physical wellbeing of Aboriginal people.

Numerous publications and reports have highlighted the poor health of Aboriginal people. Recent data from the Australian Bureau of Statistics (ABS) demonstrate that average life expectancy at birth for Aboriginal people is 67.2 years for males and 72.9 years for females; 11.5 years and 9.7 years less than the average life expectancy at birth for non-Aboriginal males and females, respectively [3]. More importantly, the health-adjusted life expectancy (estimate of the average years of equivalent ‘healthy’ life without disability) was approximately 15 years lower among Aboriginal people compared to the total Australian population [4]. A comprehensive report by Vos and colleagues (2003) [4,5] on the burden of disease and injury among Aboriginal people showed that if Aboriginal people experienced the same rate of disease burden as non-Aboriginal people, 59% of the total burden of disease in Aboriginal people could have been eliminated. The largest contributions to the health gap were from non-communicable diseases (in particular cardiovascular disease) and occurred among middle-aged (35–54 year old) Aboriginal people; 58% of the health gap occurred in people aged ≥35 years. The major risk factors suggested to have a significant influence on the disease burden were tobacco use, high body mass index (BMI), physical inactivity, high blood cholesterol and alcohol consumption. Although it is well accepted that prevention of disease through elimination of these risk factors earlier in life may be the best approach, research also needs to focus on the immediate health needs within the population, to quantify the extent to which people who are already sick and disabled are receiving appropriate health care services and social support, and how their wellbeing can best be maximised. In order to achieve this, an in-depth analysis of the health and welfare of middle-aged and older Aboriginal people and the potential barriers to care that exist is required. Moreover, improving the lives of Aboriginal elders will no doubt have a positive impact on improving the lives of younger family members and the future generation to prevent the on-going cycle of disease and disability.

Currently, detailed information on the overall health, well-being and lifestyle of middle-aged and older Aboriginal Australians from a range of socio-economic backgrounds is lacking. The majority of our knowledge comes from cross-sectional surveys [6,7] and to date, the array of sizeable longitudinal studies among Aboriginal people are limited to mostly studies among children and young adults [8,9] or targeted towards specific conditions such as diabetes [10]. Most include only Aboriginal participants, precluding direct comparisons with the general population in a number of areas. Moreover, a closer examination of the issues that are especially relevant to ageing Aboriginal people is essential in order to most appropriately target future public health initiatives. The aim of this study is to quantify socio-demographic and health risk factors and mental and physical health status among Aboriginal participants from the 45 and Up Study and to compare these with non-Aboriginal participants.

## Methods

The Sax Institute’s 45 and Up Study is a large-scale longitudinal cohort study of men and women aged 45 years and older from the general population of New South Wales (NSW), Australia that has been designed to provide reliable evidence to inform policy to support healthy ageing. NSW has the largest Aboriginal population of all the States and Territories in Australia; approximately 30% of Australia’s Aboriginal population reside in NSW [3]. The study’s baseline questionnaire included a range of questions related to socio-demographic factors, general health and social risk factor profiles and past and present medical history. Follow-up data will also be collected from the study participants by questionnaire around five years after baseline, allowing comparison with findings in the baseline survey. Links to routine sources of health data in NSW such as hospitalisations is being undertaken through data linkage [11]. Further information about the study is available at https://www.saxinstitute.org.au/our-work/45-up-study/.

### Participant recruitment

Details of participant recruitment and data collection have been reported previously [12]. Briefly, individuals aged 45 years and over were randomly selected from the Medicare Australia database (the national universal health insurance scheme), with an oversampling of regional and remote areas and individuals aged 80 years and over. Participants entered the study by completing a baseline postal questionnaire which was distributed between 1 February 2006 and 31 December 2008 and providing written consent to follow their health in the long term, through repeat questionnaires and linkage to health records. A total of 266,805 people were recruited to the study over this time. Only participants with age recorded as 45 years or greater at the time of survey and valid date of entry were included (n = 266,611). Aboriginal status was self-identified in the baseline questionnaire in response to the question: ‘Are you of Aboriginal or Torres Strait Islander Origin? With the following tick box options: 1) No 2) Yes, Aboriginal and 3) Yes, Torres Strait Islander; participants were able to select both Aboriginal and Torres Strait Islander. Of the total participants, 1746 (0.7%) identified as Aboriginal, 164 (0.06%) as Torres Strait Islander and 29 (0.01%) as both Aboriginal and Torres Strait Islander origin. Participants with missing/invalid Aboriginal status (n = 4688) were excluded. The term ‘Aboriginal’ has been used to refer to participants of Aboriginal and/or Torres Strait Islander origin unless otherwise specified, in keeping with advice from the Aboriginal Health and Medical Research Council.

The 45 and Up Study as a whole has received ethical approval from the Human Research Ethics Committee of the University of New South Wales (reference 10186). Ethical approval for the current study has been granted by the Aboriginal Health and Medical Research Council of New South Wales (reference 775/11).

### Data collection

All variables used in this study were derived from the self-reported baseline questionnaire apart from the Accessibility Remoteness Index of Australia Plus (ARIA+) score [13] and the Index of Relative Socio-economic Disadvantage (IRSD) [14] which were derived for each participant’s postcode of residence at the time of recruitment as recorded by Medicare Australia.

Socio-demographic information included age, sex, highest formal educational qualification, household annual pre-tax income, current employment status, private health insurance status, ARIA + score and IRSD. Participants were grouped into quintiles of the IRSD score. Those in quintile 1 were those that were the most disadvantaged and those in quintile 5 were those that were the least disadvantaged [14].

To assess the proportion of the Aboriginal population from NSW taking part in the 45 and Up study sample, data from the 2006 population estimates produced by the ABS were used. In order to account for differences in sampling, post-stratification estimation weights were assigned to the 45 and Up data. The post-strata were formed according to remoteness (major city, inner regional, outer regional, remote and very remote), sex and age (five year groups from 45–64 years and 65 years and over, based on the availability of ABS data).

Lifestyle and health risk factor variables included those relating to smoking, alcohol, physical activity, sedentary time, BMI and fruit and vegetable intake. Participants’ overall level of physical activity was classified according to their responses to questions on the number of weekly sessions (of any duration) of moderate and vigorous physical activity and episodes of walking for longer than 10 min, using items from the Active Australia questionnaire [15]. A weighted weekly average number of sessions was calculated for each participant by adding the total number of sessions, with vigorous activity sessions receiving twice the weighting of moderate activity or walking sessions [15]. Physical activity was classified as either sufficient physical activity (150 min of physical activity in 5 or more sessions a week) or insufficient physical activity (greater than 1 but less than or equal to 149 min), based on the guidelines from the Australian Institute of Health and Welfare (AIHW) [15]. Sedentary time was based on total daily hours of screen-time (watching television or using a computer). Self-reported weight and height measurements were used to calculate participant’s BMI, as their weight in kilograms divided by the square of their height in metres (kg/m^2^). BMI was categorized according to the World Health Organization (WHO) criteria as underweight (BMI < 18.5 kg/m^2^), overweight (25.0-29.99 kg/m^2^) and obese (BMI ≥ 30 kg/m^2^) [16]. Fruit (including fruit juice) and vegetable (including both raw and cooked vegetables) intake was assessed as servings per day and classified as adequate (≥ 2 servings of fruit and ≥ 5 servings of vegetables per day) or inadequate (less than these amounts) according to the National Health and Medical Research Council guidelines [17].

Past history of and current treatment for certain medical conditions were assessed based on the participant’s response to the questions ‘Has the doctor ever told you that you have…’ and ‘In the last month have you been treated for…”, respectively, followed by a list of conditions that the participant could select. Multiple morbidity was examined by identifying participants that had ever been diagnosed with ‘0’, ‘1-2’, ‘3-4’, ‘5-6’ and ‘7 or more’ medical conditions (participants with an invalid/missing response to any of the condition listed were excluded). Past history of operations was based on the participant’s response to the question ‘Have you had any of the following operations?’, followed by a list of common operations to choose from, including heart or coronary bypass surgery, removal of gallbladder, removal of skin cancer and hip or knee replacement.

Individuals who reported needing assistance with daily tasks because of long-term illness or disability were considered to have a major disability. Functional capacity was assessed using the Medical Outcomes Study Physical Functioning Scale [18]; a lower physical functioning score indicates more severe functional limitation [19]. The questions on the physical functioning scale ask participants about whether they are limited in their ability to perform vigorous and moderate physical activities and tasks such as: lifting shopping, climbing stairs, walking, bending, kneeling or stooping and bathing or dressing. A score is calculated where there are up to 5 missing items [18]. Functional limitation scores were classified into 5 groups: no limitation (score of 100), minor limitation (score 95–99), mild limitation (score 86–94), moderate limitation (60–84) and severe limitation (score 0–59).

Self-rated health and quality of life were based on the question, “In general, how would you rate your: Overall health? Quality of life?” followed by options of excellent, very good, good, fair and poor. Psychological distress was measured using the Kessler-10 score [20]; a scale based on 10 items used to measure non-specific psychological distress. Logical imputations were performed for missing values where there is a valid value for a similar but more severe item. For example, when the value “how often did you feel: depressed” is missing, then the value for “how often did you feel: so depressed that nothing could cheer you up” is imputed to the less severe item. The average of all non-missing items is imputed for up to one missing item, and then the final score is calculated; a higher score indicated a higher level of psychological distress. Final scores were only calculated for those participants that had a response for all ten questions after imputation as described above. Kessler-10 scores were classified into 4 groups: low psychological distress (score 10–15), moderate psychological distress (score 16–21), high psychological distress (score 22–29) and very high psychological distress (score 30 or higher) [21].

### Statistical analyses

Crude frequencies and percentages for Aboriginal and non-Aboriginal participants were tabulated for each variable of interest. Odds ratios (ORs) and 95% confidence intervals (CIs) for Aboriginal versus non-Aboriginal participants for socio-demographic, lifestyle and risk factors and medical history were estimated using unconditional logistic regression, adjusting (where appropriate) for age (as a continuous variable) and sex. For example, an OR of 3.0 for the relationship of current regular smoking among Aboriginal versus non-Aboriginal participants means that Aboriginal participants had around 3 times the likelihood of being current regular smokers than non-Aboriginal participants. The effect on the OR of additional adjustment for income, education, ARIA + and IRSD was examined to investigate the potential mediating role of these variables. For continuous variables a Student’s t-test was used to examine statistically significant differences between the Aboriginal participants compared to the non-Aboriginal participants. Within population groups, differences between males and females were also examined. For continuous variables, a multiple comparison test using the least squares means method with Bonferroni adjustment was used taking into account age, sex and Aboriginal status. The proportion of Aboriginal and non-Aboriginal participants ‘ever diagnosed with heart disease, high blood pressure, diabetes, stroke, depression and asthma’ was calculated stratified by age group category; whether age was a significant interacting factor was assessed using logistic regression. All statistical analyses were undertaken using SAS software version 9.3 (SAS Institute Inc, Cary, NC, USA). All statistical tests were two-sided, with a significance level of P < 0.05, except when Bonferroni adjustment was used.

## Results

Among the 266,611 participants examined in the current study, there were 1939 (0.7%) participants who identified as Aboriginal and/or Torres Strait Islander origin (1746 (0.7%) as Aboriginal, 164 (0.06%) as Torres Strait Islander and 29 (0.01%) as both Aboriginal and Torres Strait Islander origin) and 4688 (1.8%) participants whose Aboriginal status was unknown; participants with an unknown Aboriginal status were excluded. Compared to the general Aboriginal population, younger individuals (45–49 years) and those living in major cities were under-represented in the 45 and Up study (Table 1). Those living in inner and outer regional and remote areas were over-represented. Participants of Torres Strait Islander origin (including those of both Torres Strait Islander and Aboriginal origin) were significantly older than the participants of Aboriginal origin alone, they had a lower mean body mass index and were less likely to be current (OR 0.5, 95% CI 0.4-0.7) or former smokers (OR 0.5, 0.3-0.8). Education and income did not differ significantly between these two participant groups. As stated previously, the term “Aboriginal” will be used to refer to all Aboriginal and/or Torres Strait Islander participants for the remainder of the manuscript.

**Table 1:** Proportions of 45 and Up Aboriginal participants weighted for sex, age and remoteness with the 2006 Australian Bureau of Statistics Census data for New South Wales

	**n**	**Crude% (CI)**	Weighted*% (CI)
**Age**			
45-49	370	19 (17–21)	28 (26–31)
50-54	489	25 (23–27)	22 (20–24)
55-59	373	19 (17–21)	17 (16–19)
60-64	267	14 (12–15)	12 (10–14)
65+	440	23 (21–25)	20 (18–22)
**Remoteness (ARIA +)**			
Major city	598	31 (29–33)	43 (40–45)
Inner regional	685	35 (35–37)	27 (25–29)
Outer regional	519	27 (25–29)	25 (22–27)
Remote	115	6 (5–7)	5 (4–6)
Very remote	22	1 (0.7-2)	0.7 (0.4-1)
**Sex**			
Male	838	43 (41–45)	47 (45–50)
Female	1101	56 (55–58)	53 (50–55)

*Weighted for sex, age and remoteness with the 2006 Australian Bureau of Statistics Census data for New South Wales.

### Socio-demographic factors

Compared to non-Aboriginal participants, Aboriginal participants were younger on average (average age 58 versus 63 years) (Table 2). Adjusting for age and sex, compared to non-Aboriginal participants, Aboriginal participants were more likely to have: no formal educational qualifications (OR = 6.2, 95% CI 5.3-7.3), a work status of “disabled or sick” (4.6, 3.9-5.3) or “unemployed” (3.7, 2.9-4.6) and an annual pre-tax household income of less than $20,000 versus greater than or equal to $70,000 (5.8, 5.0-6.9).

**Table 2:** Socio-demographic characteristics of the Aboriginal and the non-Aboriginal participants

	**Aboriginal participants**	**Non-Aboriginal participants**	**Odds ratio for Aboriginal vs non-Aboriginal participants**
	**(n = 1939)**	**(n = 259984)**	
	**% (n)**	**% (n)**	**Adjusted for age and sex, where appropriate**
**Sex**			
Male	43 (838)	46 (120735)	1.0
Female	57 (1101)	54 (139249)	1.1 (1.0–1.2)
**Age**			
*mean* ± *SD*	57.9 ± 9.2^	62.7 ± 11.1	–
45-49	19 (370)	11 (29876)	1.0
50-59	44 (862)	33 (86685)	0.8 (0.7–0.9)
60-69	24 (458)	28 (73696)	0.5 (0.4–0.6)
70-79	9 (184)	16 (42244)	0.4 (0.3–0.4)
80+	3 (65)	11 (27483)	0.2 (0.1–0.3)
**Highest qualification**			
None	29 (557)	11 (29796)	6.2 (5.3–7.3)
School/Intermediate/High School Certificate	28 (543)	32 (82797)	1.8 (1.5–2.0)
Trade/apprenticeship/certificate/diploma	26 (510)	32 (83071)	1.6 (1.4–1.8)
University degree or higher	14 (266)	23 (60631)	1.0
**Marital status**			
Married/De facto/Living with partner	60 (1169)	75 (193742)	1.0
Single	13 (249)	6 (14628)	2.7 (2.3–3.1)
Widowed	7 (144)	9 (22320)	1.4 (1.1–1.8)
Divorced/Separated	18 (355)	11 (27811)	1.6 (1.3–1.9)
**Work status**			
Full-time/Part-time paid work	34 (655)	31 (81282)	1.0
Looking after home/family	8 (153)	7 (18103)	1.4 (1.2–1.8)
Retired	22 (424)	36 (93653)	1.2 (1.0–1.5)
Disabled/sick	14 (262)	3 (9026)	4.6 (3.9–5.3)
Unemployed	5 (89)	1 (3815)	3.7 (2.9–4.6)
Other	17 (327)	20 (52122)	0.9 (0.8–1.1)
**Household annual pre-tax income**			
<$20,000	31 (597)	19 (50629)	5.8 (5.0–6.9)
$20,000-$39,999	17 (327)	18 (45596)	2.7 (2.2–3.2)
$40,000-$69,999	14 (276)	18 (46300)	1.6 (1.3–1.9)
≥$70,000	14 (281)	24 (61983)	1.0
**Health insurance**			
None	24 (458)	15 (39465)	1.0
Private (with or without extras)	28 (551)	54 (140261)	0.4 (0.3–0.4)
Health Care Card	43 (835)	27 (70567)	1.8 (1.6–2.0)
Department of Veteran Affairs Card	1 (17)	2 (4973)	0.8 (0.5–1.3)
**Remoteness (ARIA+)**			
Major City	31 (598)	45 (117325)	1.0
Inner Regional	35 (685)	35 (91344)	1.4 (1.3–1.6)
Outer Regional	27 (519)	18 (46049)	2.1 (1.9–2.4)
Remote/Very Remote	7 (137)	2 (5065)	4.9 (4.1–5.9)
**Index of relative social disadvantage (IRSD)**			
1st Quintile (most disadvantaged)	36 (706)	20 (52058)	5.7 (4.7–6.9)
2nd Quintile	23 (450)	20 (52986)	3.5 (2.9–4.3)
3rd Quintile	19 (366)	20 (52378)	2.9 (2.3–3.5)
4th Quintile	15 (293)	20 (50752)	2.3 (1.9–2.9)
5th Quintile (least disadvantaged)	6 (124)	20 (51594)	1.0

Percentages may not equal to 100 due to missing/invalid data.

^ Significantly different to non-Aboriginal group (P < 0.05, using Student’s t-test).

### Health risk factors

Forty percent of the Aboriginal participants reported never being a regular smoker and 44% reported having none or less than one alcoholic drink per week (Table 3). Average BMI among the Aboriginal participants was 29 kg/m^2^; with approximately a third of the Aboriginal cohort classified as obese. Major disparities in health risk factors between Aboriginal and non-Aboriginal participants were found in current regular smoking and BMI. Overall, 21% of Aboriginal and 7% of non-Aboriginal participants reported being current regular smokers and the age and sex adjusted OR (95% CI) for current smoking was 3.6 (3.2-4.1) for Aboriginal compared to non-Aboriginal participants. Aboriginal participants were significantly more likely to be overweight (OR = 1.4 (1.3-1.6)) or obese (OR = 2.5 (2.2-2.8)) compared to non-Aboriginal participants. Although, the average number of alcoholic drinks per week was not significantly different between Aboriginal and non-Aboriginal participants; after adjusting for age, sex, income and education, Aboriginal participants were less likely to consume 1–4, 5–7, 8–14 alcoholic drinks per week. There was no significant difference in Aboriginal status for consumption of 15 or more alcoholic drinks per week.

**Table 3:** Health risk factors, physical disability and functional health limitations among Aboriginal and non-Aboriginal participants by sex

	**Aboriginal participants**	**Non-aboriginal participants**	**Odds ratio for Aboriginal participants vs non-Aboriginal participants**	
	**(n = 1939)**	**(n = 259984)**		
	**% (n)**	**% (n)**	**Adjusted for age and sex**	**Adjusted for age, sex, income**, **education**
**Regular tobacco smoker**				
Never	40 (772)	56 (144910)	1.0	1.0
Current	21 (416)	7 (18595)	3.6 (3.2–4.1)	2.4 (2.0–2.8)
Former	38 (738)	37 (95720)	1.5 (1.4–1.7)	1.4 (1.2–1.6)
**Alcohol (number of drinks per week)**				
mean ± SD	6.8 ± 12.5	7.0 ± 9.7	–	–
0	44 (851)	32 (83829)	1.0	1.0
1-4	16 (303)	20 (51897)	0.5 (0.4–0.6)	0.6 (0.5–0.7)
5-7	10 (188)	14 (37332)	0.5 (0.4–0.6)	0.6 (0.5–0.8)
8-14	12 (234)	17 (45215)	0.5 (0.4–0.5)	0.6 (0.5–0.8)
≥15	14 (269)	14 (36668)	0.6 (0.5–0.7)	0.9 (0.7–1.0)
Body mass index*				
mean ± SD	28.9 ± 5.9^	26.9 ± 4.9	–	–
Underweight (<18.5)	2 (33)	1 (3287)	2.6 (1.8–3.7)	2.0 (1.3–3.1)
Healthy weight (18.5–24.99)	21 (403)	34 (88834)	1.0	1.0
Overweight (25–29.99)	32 (627)	37 (95232)	1.4 (1.3–1.6)	1.5 (1.3–1.7)
Obese (≥30)	33 (647)	21 (53504)	2.5 (2.2–2.8)	2.1 (1.8–2.5)
Physical activity (sessions/week)*				
mean ± SD	12.0 ± 19.6^	10.8 ± 14.8	–	–
Sufficient physical exercise	64 (1248)	75 (195781)	1.0	1.0
Insufficient physical exercise	36 (691)	25 (64203)	1.9 (1.8–2.1)	1.4 (1.3–1.6)
**Sedentary screen time (hours/day)**				
mean ± SD	4.4 ± 2.8^	4.2 ± 2.5	–	––
0-1	7 (130)	6 (16601)	1.2 (1.0–1.5)	1.1 (0.9–1.4)
2-3	32 (625)	38 (99640)	1.0	1.0
4-5	29 (566)	30 (78064)	1.2 (1.1–1.4)	1.3 (1.1–1.4)
6-7	12 (229)	10 (26615)	1.4 (1.2–1.6)	1.3 (1.1–1.6)
≥8	13 (245)	11 (29073)	1.1 (1.0–1.3)	1.3 (1.1–1.5)
**Vegetable intake (serves/day)**				
mean ± SD	4.0 ± 2.9	3.9 ± 2.7	–	–
Less than 5 serves	63 (1228)	66 (172074)	1.0	1.0
5 or more serves	32 (621)	31 (81721)	1.1 (1.0–1.2)	1.1 (1–1.2)
Fruit intake (serves/day)*				
mean ± SD	2.5 ± 2.2*	2.6 ± 1.8	–	–
Less than 2 serves	30 (576)	25 (63884)	1.0	1.0
2 or more serves	63 (1229)	72 (187260)	0.8 (0.7–0.8)	0.9 (0.8–1.0)
**Physical disability**				
Does not need help with daily tasks	86 (1543)	94 (234089)	1.0	1.0
Needs help with daily tasks	14 (245)	6 (13855)	3.9 (3.4–4.5)	2.6 (2.2–3.1)
**Health limitations**				
No functional limitation	23 (455)	30 (77765)	1.0	1.0
Minor functional limitation	16 (314)	25 (65415)	1.0 (0.8–1.1)	1.0 (0.9–1.2)
Moderate functional limitation	21 (404)	22 (56248)	1.8 (1.6–2.1)	1.7 (1.4–2.0)
Severe functional limitation	25 (486)	14 (35481)	4.7 (4.1–5.4)	2.9 (2.4–3.4)
**Regularly care for sick/disabled family member or friend**				
No	77 (1489)	85 (221463)	1.0	1.0
Yes –Part-time	9 (165)	7 (18444)	1.3 (1.1–1.5)	1.3 (1.1–1.6)
Yes –Full-time	9 (170)	4 (10805)	2.7 (2.3–3.1)	1.8 (1.5–2.2)

Percentages may not equal to 100 due to missing/invalid data.

^Significant difference between Aboriginal group and non-Aboriginal group (P < 0.05, as a result of Student’s t-test).

*Significant difference between Aboriginal and non-Aboriginal group (P < 0.05, as a result of a multiple comparison test adjusting for age and sex).

### Physical health, disability and psychological distress

**Figure 1:**
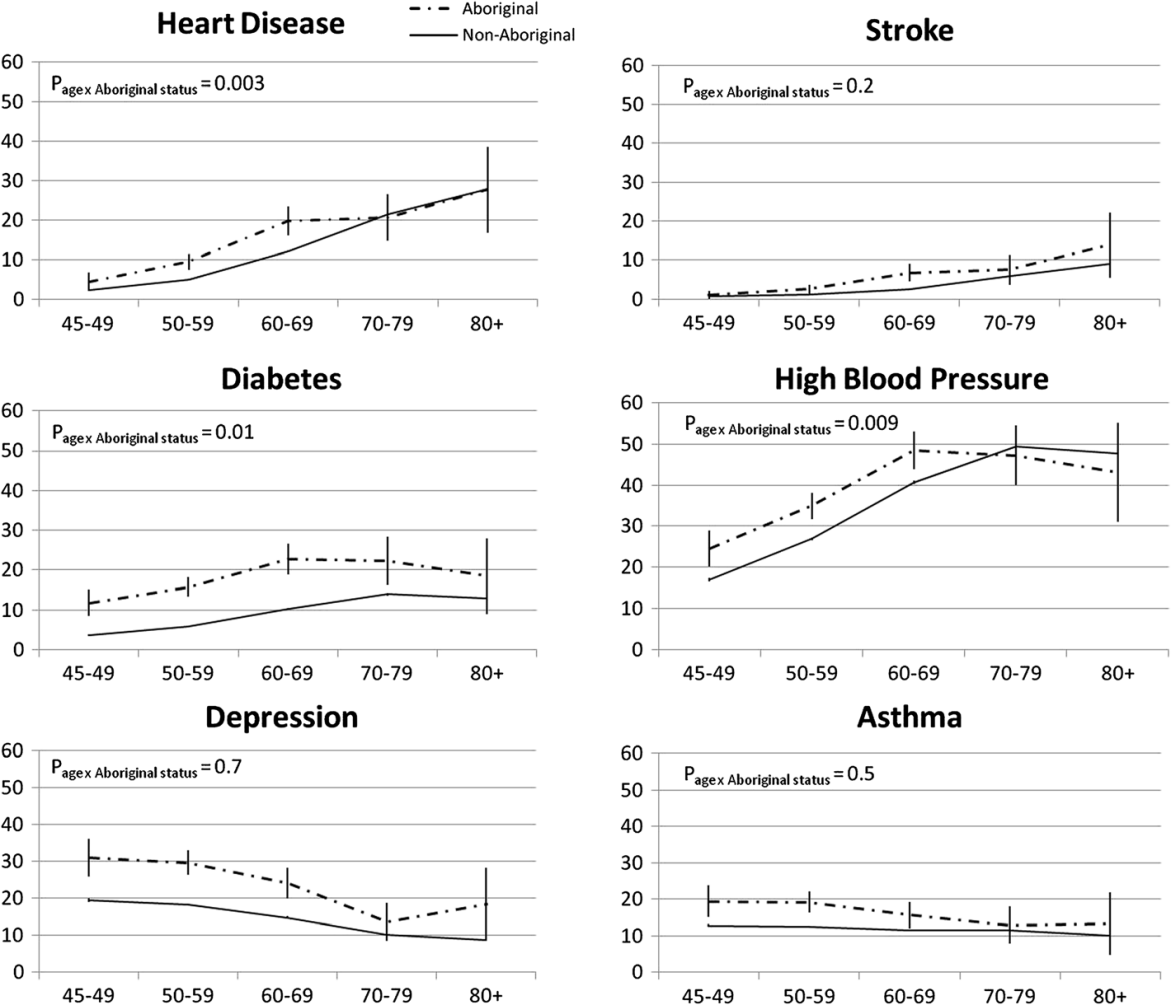
**History of ‘ever being diagnosed’ with heart disease, stroke, diabetes, high blood pressure, depression and asthma by age group in Aboriginal and non-Aboriginal participants.** Aboriginal participants are represented with the dashed black line and non-Aboriginal participants are represented with the solid black line (units are percentages with confidence intervals). There was a significant interaction between age and Aboriginal status for high blood pressure, heart disease and diabetes (P < 0.05).

The most common medical condition that Aboriginal participants had ‘ever been diagnosed with’ and were ‘currently being treated for’ was high blood pressure (38% and 36%, respectively) (Table 4). 23% of Aboriginal and 13% of non-Aboriginal participants reported ever having been diagnosed with depression. A significantly higher proportion of Aboriginal participants had ‘ever been diagnosed’ with high blood pressure, stroke, diabetes, heart disease, depression, and asthma compared to non-Aboriginal participants in all age groups (Figure 1, Table 4). Aboriginal participants were less likely to have a past history of non-melanoma skin cancer compared to non-Aboriginal participants and there was no significant difference for past history of melanoma, prostate cancer and breast cancer. Overall, 16% of Aboriginal and 19% of non-Aboriginal participants reported never having been diagnosed with any of the conditions listed. The proportion of participants diagnosed with multiple conditions was higher among the Aboriginal participants compared to the non-Aboriginal participants; fully adjusted ORs (95% CIs) for ever being diagnosed with 5–6 medical conditions and 7 or more conditions was 2.6 (2.1-3.4) and 3.8 (2.6-5.6), respectively for Aboriginal participants compared to non-Aboriginal participants.

**Table 4:** Medical and surgical history of Aboriginal and non-Aboriginal participants by sex

	**Aboriginal participants**	**Non-aboriginal participants**	**Odds ratio for Aboriginal participants vs non-Aboriginal participants**	
	**(n = 1939)**	**(n = 259984)**		
	**% (n)**	**% (n)**	**Adjusted for age and sex**	**Adjusted for age**, **sex**, **income**, **education**
***History of ***^***a***^				
Heart Disease	13 (246)	12 (30848)	1.5 (1.3–1.7)	1.5 (1.3–1.8)
High Blood Pressure^b^	38 (729)	36 (92403)	1.3 (1.2–1.4)	1.4 (1.2–1.5)
Stroke	4 (80)	3 (8090)	1.9 (1.5–2.4)	1.8 (1.4–2.3)
Diabetes	17 (336)	9 (23048)	2.5 (2.2–2.8)	2.1 (1.8–2.4)
Thrombosis	7 (132)	5 (11939)	1.7 (1.4–2.0)	1.7 (1.3–2.1)
Asthma*	15 (292)	10 (26371)	1.5 (1.4–1.7)	1.4 (1.2–1.6)
Depression*	23 (441)	13 (33637)	1.8 (1.6–2.0)	1.6 (1.4–1.8)
Anxiety*	14 (269)	8 (21796)	1.6 (1.4–1.9)	1.4 (1.2–1.6)
Melanoma	5 (104)	5 (14215)	1.2 (1.0–1.4)	1.2 (1.0–1.5)
Skin Cancer (excluding melanoma)	16 (305)	26 (66594)	0.7 (0.6–0.8)	0.8 (0.7–0.9)
Prostate Cancer (males)	5 (44)	6 (7609)	1.2 (0.9–1.7)	1.4 (0.9–1.9)
Enlarged Prostate (males)	10 (85)	17 (20540)	0.8 (0.7–1.0)	0.9 (0.7–1.2)
Breast Cancer (females)	5 (55)	5 (7521)	1.0 (0.8–1.3)	1.2 (0.9–1.7)
Other Cancer	8 (149)	6 (16430)	1.5 (1.3–1.7)	1.3 (1.1–1.6)
Parkinson’s disease	0.5 (10)	0.6 (1617)	1.3 (0.7–2.2)	1.1 (0.5–2.3)
None of these	16 (305)	19 (50002)	0.6 (0.6–0.7)	0.7 (0.6–0.8)
***Multiple morbidity *( *No *. *of conditions***) ^**c**^				
0	25 (493)	27 (71399)	1.0	1.0
1-2	50 (961)	52 (135344)	1.2 (1.1–1.4)	1.2 (1.1–1.4)
3-4	18 (357)	17 (43772)	1.8 (1.5–2.1)	1.6 (1.4–1.9)
5-6	5 (96)	3 (7932)	3.1 (2.5–3.9)	2.6 (2.1–3.4)
7 or more	2 (32)	0.6 (1537)	4.8 (3.3–7.0)	3.8 (2.6–5.6)
***Currently treated for ***^***a***^				
Heart attack or angina	4 (86)	3 (6655)	2.9 (2.3–3.6)	2.3 (1.8–3.0)
Other heart disease	3 (63)	3 (7179)	1.7 (1.3–2.2)	1.5 (1.1–2.0)
High blood pressure^b^	28 (534)	24 (63149)	1.5 (1.4–1.7)	1.5 (1.3–1.6)
High blood cholesterol	16 (311)	15 (39378)	1.3 (1.1–1.4)	1.2 (1.1–1.4)
Blood clotting problems	3 (51)	2 (4934)	1.9 (1.5–2.6)	1.3 (0.9–1.9)
Asthma	9 (179)	5 (12083)	2.2 (1.9–2.6)	1.8 (1.5–2.2)
Osteoarthritis	10 (189)	8 (20868)	1.6 (1.4–1.9)	1.5 (1.2–1.8)
Thyroid problems	6 (124)	5 (12911)	1.4 (1.2–1.7)	1.4 (1.1–1.7)
Osteoporosis or low bone density	5 (89)	6 (14848)	1.1 (0.9–1.4)	1.0 (0.8–1.3)
Depression or anxiety	16 (44)	8 (2946)	2.0 (1.5–2.8)	1.5 (1.1–2.3)
Cancer	3 (61)	3 (7229)	1.5 (1.1–1.9)	1.4 (1.1–1.9)
None of these	35 (676)	43 (112656)	0.6 (0.5–0.6)	0.7 (0.6–0.8)
***Past operations ***^***a***^				
Heart or coronary artery bypass surgery^d^	6 (114)	6 (15320)	1.6 (1.3–1.9)	1.3 (1.0–1.7)
Gallbladder removed	12 (230)	10 (26280)	1.4 (1.2–1.6)	1.3 (1.1–1.6)
Removal of skin cancer	18 (342)	27 (69157)	0.7 (0.6–0.8)	0.8 (0.7–0.9)
Hip replacement	2 (44)	3 (8368)	1.1 (0.8–1.5)	1.3 (0.9–1.8)
Knee replacement	4 (75)	4 (11034)	1.4 (1.1–1.8)	1.4 (1.1–1.8)
Hysterectomy (females)	35 (387)	28 (39038)	1.6 (1.4–1.8)	1.5 (1.3–1.8)
Bilateral oophorectomy (females)	14 (154)	10 (13572)	1.8 (1.5–2.1)	1.8 (1.5–2.2)
Tubal ligation	34 (373)	26 (36729)	1.4 (1.2–1.6)	1.4 (1.2–1.6)
Repair of prolapsed bladder, womb or bowel (females)	15 (162)	11 (15750)	1.7 (1.4–2.0)	1.6 (1.3–1.9)
Vasectomy (males)	21 (174)	24 (29563)	0.6 (0.5–0.8)	0.8 (0.6–0.9)
Part of prostate removed (males)	2 (20)	6 (6953)	0.7 (0.5–1.1)	0.9 (0.5–1.4)
Whole of prostate removed (males)	3 (21)	4 (4347)	1.0 (0.7–1.6)	1.2 (0.7–1.9)

Percentages may not equal to 100 due to missing/invalid data.

*Not included in Version 1 questionnaire (excludes 37,000 participants).

^a^self reported conditions.

^b^excluding pregnancy related hypertension.

^c^excludes participants with a invalid/missing response to any of the conditions listed.

^d^includes grafts, balloons and stents.

The prevalence of high blood pressure, heart disease and diabetes stratified by age group varied significantly between Aboriginal and non-Aboriginal participants (P[interaction] = 0.009, P[interaction] = 0.003 and P[interaction] = 0.01, respectively) (Figure 1). Prevalence of stroke, depression and asthma according to age did not differ significantly between Aboriginal participants and non-Aboriginal participants.

The most common type of surgical procedure among both the Aboriginal and non-Aboriginal participants was removal of skin cancer. Adjusting for age, sex and other socio-demographic characteristics, Aboriginal participants were significantly more likely than non-Aboriginal participants to have had their gallbladder removed or to have a knee replacement. Compared to non-Aboriginal women, Aboriginal women were significantly more likely to have had: bilateral oophorectomy, repair of prolapsed bladder, womb or bowel, hysterectomy and tubal ligation.

Aboriginal participants were more likely to have physical disabilities compared to non-Aboriginal participants; fourteen percent of Aboriginal participants reported having a major physical disability compared to 6% of non-Aboriginal people. Compared to non-Aboriginal participants, the adjusted OR for major physical disability was 2.6 (2.2-3.1) for Aboriginal participants. Similarly, the adjusted OR was 2.9 (2.4-3.4) for severe functional limitation for Aboriginal participants compared to non-Aboriginal participants. Aboriginal participants were also more likely to be part-time or full-time carers for a sick or disabled family member or friend compared to non-Aboriginal participants; overall 9% of Aboriginal participants were full-time carers. Compared to non-Aboriginal participants, the adjusted OR for reporting poor health and quality of life was 3.6 (2.8-4.6) and 3.4 (2.6-4.4), respectively for Aboriginal participants. In regards to psychological distress, the adjusted ORs for high and very high psychological distress for Aboriginal participants compared to non-Aboriginal participants were 1.8 (1.5-2.2) and 2.4 (2.0-3.0), respectively (Table 5). Furthermore, Aboriginal participants were also more likely than non-Aboriginal participants to have ‘cut down on time spent on work or other activity’, have achieved ‘less than they would have liked to’ and to report doing ‘work or other activities less carefully’, due to emotional problems (Table 5).

**Table 5:** **Self rated health**, **quality of life and psychological distress among Aboriginal and non-Aboriginal participants by sex**

	**Aboriginal participants**	**Non-Aboriginal participants**	**Odds ratio for Aboriginal participants vs non-Aboriginal participants**	
	**(n = 1939)**	**(n = 259984)**		
	**% (n)**	**% (n)**	**Adjusted for age and sex**	**Adjusted for age**, **sex**, **income**, **education**
**Self rated health**				
Excellent/Very good	33 (642)	51 (131507)	1.0	1.0
Good/fair	54 (1055)	44 (114354)	2.2 (2.0–2.4)	1.6 (1.5–1.8)
Poor	7 (135)	2 (5341)	6.5 (5.3–7.8)	3.6 (2.8–4.6)
**Self rated quality of life**				
Excellent/Very good	39 (757)	58 (151391)	1.0	1.0
Good/fair	46 (887)	35 (91261)	2.3 (2.1–2.6)	1.6 (1.4–1.7)
Poor	5 (105)	2 (4057)	6.3 (5.1–7.7)	3.4 (2.6–4.4)
**Level of psychological distress (based on Kessler 10 score)**				
Low	50 (971)	68 (177844)	1.0	1.0
Moderate	16 (319)	14 (36593)	1.5 (1.3–1.7)	1.3 (1.1–1.5)
High	10 (191)	5 (12380)	2.5 (2.1–2.9)	1.8 (1.5–2.2)
Very High	8 (150)	2 (4957)	4.9 (4.1–5.8)	2.4 (2.0–3.0)
**Problems with work or daily activities due to emotional problems**				
Cut down on amount of time spent on work or other activities	23 (408)	11 (27242)	2.3 (2.1–2.6)	1.9 (1.7–2.2)
Achieved less than would have liked to	42 (740)	29 (71525)	1.8 (1.6–1.9)	1.6 (1.5–1.8)
Did work or other activities less carefully	23 (391)	13 (30660)	1.9 (1.7–2.1)	1.7 (1.5–1.9)

Percentages may not equal to 100 due to missing/invalid data.

Although significant associations remained, further adjustment for income and education attenuated the odds ratios for the outcomes examined, suggesting that these are mediating factors. For health risk factors, full adjustment attenuated the OR by 46% for current regular smoking status and by 27% for the risk of being obese. In regards to previous medical conditions, full adjustment attenuated the OR from 11% to 66% for certain conditions or did not change the OR in others. The mediating role of formal education level and income was most evident for high and very high psychological distress (47% & 64%), severe functional health limitations (49%), major physical disability (45%) and full-time carer status (53%).

## Discussion

The findings of this study have shown that Aboriginal participants of the 45 and Up study are more likely to be younger, be socially disadvantaged, have a greater number of health risk factors, suffer from a range of medical conditions, have major physical disability, have severe functional health limitations and have high or very levels of psychological distress compared to the non-Aboriginal participants.

Similar to previous reports [6,7], the largest disparity in health risk factors between the Aboriginal participants and the non-Aboriginal participants was found in current smoking rates and in BMI. Twenty-one percent of the Aboriginal participants were current regular smokers compared to 7% among non-Aboriginal participants. Although the reported prevalence of smoking among younger Aboriginal people is much higher [6,7,22], the relatively high proportion of smokers among ageing Aboriginal people in this study is of particular concern, due to their higher absolute risk of conditions such as cardiovascular disease. The proportion of non-drinkers of alcohol was greater among Aboriginal participants compared to non-Aboriginal participants. However, there was no significant difference between Aboriginal and non-Aboriginal participants in terms of those consuming ≥15 drinks per week, suggesting that those that did consume alcohol were more likely to be heavy drinkers.

The high proportion of Aboriginal participants of the 45 and Up study classified as ‘overweight’ or ‘obese’ was similar to previous reports [6,7]. Aboriginal participants were twice as likely to be obese compared to non-Aboriginal participants. Although investigation of factors related to the high proportions of overweight and obese participants is beyond the scope of this paper, we did find that Aboriginal participants were less likely to have sufficient physical exercise and also had more sedentary time compared to non-Aboriginal participants. Further investigation of the relationship between diet and BMI may also elucidate some important factors associated with the high prevalence of obesity among Aboriginal participants of the 45 and Up study.

In keeping with the existing evidence, Aboriginal participants were more likely than non-Aboriginal participants to have ever been diagnosed with diabetes, stroke, depression, anxiety, heart disease and high blood pressure and were also more likely to have been diagnosed with multiple conditions during their lifetime. Of note, 16% and 35% of the Aboriginal participants reported not ever being diagnosed and not currently being treated for any of the conditions listed, respectively; hence, it is important to further investigate these individuals in future studies and determine the factors that are potentially associated with their ‘disease-free’ state. Highest level of formal education and income level were found to have the largest influence on the risk of Aboriginal-attributed risk of diabetes; adjustment for these factors attenuated the odds ratio for Aboriginal versus non-Aboriginal participants by 27% from 2.5 to 2.1. It is also important to note that history of ever being diagnosed with age-related chronic diseases such as diabetes, heart disease and high blood pressure peaked a decade earlier (60–69 years compared to 70–79 years) among Aboriginal participants compared to non-Aboriginal participants which reflects the younger age distribution of the Aboriginal population and the earlier onset of diseases among Aboriginal people.

A number of previous reports and studies have described the high levels of psychological distress among Aboriginal people [6,7,22,23]. Furthermore, mental health disorders have been shown to be a significant contributor to the high burden of disease among the Aboriginal population [5]. In the current study, 23% of Aboriginal participants had ever been diagnosed with depression during their lifetime. In addition, Aboriginal participants were also more likely than non-Aboriginal participants to have moderate, high and very high levels of psychological distress (with the disparity increasing with increasing severity) and were more likely to report that emotional problems had an impact on work and other activities. Also, a national report published by the AIHW showed that Aboriginal people with high/very levels of psychological distress were more likely to report fair/poor health and were also more likely to be daily smokers and consume high levels of alcohol [22].

Compared to non-Aboriginal participants, Aboriginal participants had five times the risk of reporting a work status of ‘disabled/sick,’ three times the risk of reporting a major physical disability (requiring help with daily tasks) and three times the risk of reporting severe physical functional limitations. This finding is in accordance to national data from the AIHW which show that the need for assistance with core activities was three times higher among Indigenous Australians compared to non-Indigenous Australians and was greatest in the 45–54 and 55–64 year age groups. Additionally, Aboriginal people were significantly more likely to be full-time carers for a sick or disabled family member or friend compared to non-Aboriginal people. Taken together, these indicators suggest that physical disability is a major problem among older Aboriginal people and their communities.

Although strong associations remained between Aboriginality and measures of health risk, ill health, psychological distress and disability in the fully adjusted models shown here, many of the associations examined were attenuated by around 11% to 66% following additional adjustment for highest level of formal education attained and income level. This suggests that lower levels of income and formal education are contributing to these associations, however there remains considerable proportions of the observed differences between Aboriginal and non-Aboriginal participants that are not explained by these factors.

### Study strengths and limitations

The 45 and Up study includes data on a wide range of health-related factors from a large sample of middle-aged and older Aboriginal and non-Aboriginal participants from NSW. The inclusion of both Aboriginal and non-Aboriginal participants within the same cohort allows direct quantitative comparisons between the two groups. The current report is based on a cross-sectional survey of self-reported data; therefore, there are inherent limitations in determining causal relationships. The response rate of all participants (Aboriginal and non-Aboriginal) is estimated to be 18%. This means that the absolute prevalence reported here should be interpreted with caution. However, previous work has shown that internal comparisons, particularly the observed odds ratios, are likely to be reliable and generalisable more broadly [24]. Moreover, the findings are comparable to previously conducted studies, suggesting that the potential biases are not having a large impact on the study results.

It was not possible to obtain specific response rates among Aboriginal participants since data on Aboriginal identification was available only among responders to the questionnaire. It is to be noted that the ability to complete the questionnaire in English may have preferentially selected participants with a higher level of formal education and of higher socio-economic status, compared to the general population.

An unweighted comparison of the 45 and Up study Aboriginal population to the NSW Indigenous population aged over 45 years (2006 NSW Census data available from the Australian Bureau of Statistics (ABS)) showed that the proportion of Aboriginal males and female study participants with a university degree or higher qualification was 2–3 times that of the Aboriginal people in the general population (4% compared to 12% in males (p < 0.0001), and 7% compared to 15% in females (p < 0.0001)). A higher proportion of study participants were married or in a de facto relationship than the general population (p < 0.0001), and the proportion of people needing help with core activities in the 65 and over age group was significantly lower in our sample (18% compared to 25%) (p = 0.002), but not across all relevant age groups aged 45 years and over (14% in both) (p = 0.65). The lower levels of major disability in older people are likely due to the method of data collection, as the 45 and Up Study required participants to complete the questionnaire themselves while the Census could be completed by another member of the household. Therefore, overall, it is possible that the health issues described in the study may be an underestimate of the true burden among Aboriginal people of NSW. However, even with the possibility of this bias, the large disparity observed in comparison to non-Aboriginal participants gives an indication of the levels of disease and disability among Aboriginal people.

Potential issues relating to data quality for the Aboriginal variable should also be considered when interpreting the results. The proportion of the cohort identifying as Aboriginal is low within the cohort, so the variable is more likely to be affected by issues such as random misclassification (for example, due to data entry errors or misunderstanding of the questionnaire item). Any such misclassification is likely to bias results towards the null, so would result in an underestimate of the underlying risks and relative risks. A program of work to validate the Aboriginal identification variables used in the 45 and Up Study is underway. Currently, there is a lack of clinical data available for this cohort, however, as the study progresses, data available will increase.

### Study implications and areas for future research

Among the participants of this study, not only are Aboriginal people more likely to have a greater number of health risk factors and major medical conditions compared to non-Aboriginal people, high levels of physical disability and psychological distress also co-exist. A significant portion of the increased risk among Aboriginal participants was explained by level of formal education attained and income level. Educational attainment is an important indicator of socio-economic status since it remains stable throughout the life course after young adulthood and is usually established prior to employment and income level [25]. Hence, promoting formal educational qualifications among Aboriginal people during their younger years which may potentially increase employment opportunities and thereby income level later in life may have a beneficial impact on long-term mental and physical health. However, the findings relating to income level should be interpreted more cautiously, since ill health can impact on income and hence be an effect, rather than a cause of illness.

Further examination of the factors associated with increased morbidity among the 45 and Up Aboriginal participants and follow-up data will provide more insight into ways of preventing the high morbidity and premature mortality among Aboriginal people. Findings of this study also suggest that greater attention may be needed towards addressing social inequalities in health in Australia and providing appropriate mental health services and disability support for Aboriginal people. Given that Aboriginal participants of this study were significantly younger than the non-Aboriginal participants, it is also important to tailor health policies and support services for ageing Aboriginal people in accordance to this younger age distribution.

## Conclusions

The current study has provided an overview of the socio-demographic and health characteristics of middle-aged and older Aboriginal participants of the 45 and Up Study and the large disparity between Aboriginal and non-Aboriginal participants. In particular, among Aboriginal people there are greater levels of physical disability, functional health limitations and psychological distress; these are key areas for future public health policy and research.

## Abbreviations

ABS: Australian Bureau of Statistics; AIHW: Australian Institute of Health and Welfare; ARIA+: Accessibility remoteness index of Australia plus; BMI: Body mass index; CI: Confidence intervals; IRSD: Index of relative socio-economic disadvantage; NSW: New South Wales; OR: Odds ratio; WHO: World Health Organization.

## Competing interests

The authors have no competing interests to declare.

## Authors’ contributions

LG contributed to the data analyses and interpretation of results and drafted the initial manuscript. BM was involved in study conception and design, data analyses and drafting the manuscript. EB was involved in the design of the study, acquisition of data, data analyses and interpretation of results and in drafting the manuscript. GJ participated in data analyses and interpretation of results and in drafting the manuscript. AW and BR were involved in drafting and revising the manuscript. SJE was involved in the conception and design of the study, data analyses and interpretation of results and in drafting the manuscript. All authors were involved in revising the manuscript critically for important intellectual content and have given final approval of the final version of the manuscript.

## Pre-publication history

The pre-publication history for this paper can be accessed here:

http://www.biomedcentral.com/1471-2458/13/661/prepub
